# The Relationship Between Personality and Perceived Mental Fatigability

**DOI:** 10.1093/geroni/igaa057.2832

**Published:** 2020-12-16

**Authors:** Hannah Allen, Theresa Gmelin, Stephen Smagula, Robert Boudreau, Jane Cauley, Nancy Glynn

**Affiliations:** 1 University of Pittsburgh Graduate School of Public Health, Pittsburgh, Pennsylvania, United States; 2 University of Pittsburgh, Pittsburgh, Pennsylvania, United States

## Abstract

Several personality traits are known to be protective against global fatigue, however perceived mental fatigability (PMF, Pittsburgh Fatigability Scale 0-50) specifically measures an individual’s susceptibility to cognitive tiredness and is associated with mobility decline. We assessed whether optimism, conscientiousness, goal reengagement and goal disengagement contributed to greater PMF in 1,812 men (mean±SD age 84.4±4.2 years, 90.4% white) in the Osteoporotic Fractures in Men Study 4th visit (2014-2016). Covariates included demographic, psychological/behavioral factors, health conditions, physical activity and function. Prevalence of higher PMF (score ≥13) was 25% (n=448). In a covariate-adjusted regression model, each SD lower conscientiousness and lower optimism were associated with 0.93 and 0.61 SDs greater PMF, each p<0.01. Goal disengagement and goal reengagement were not associated with PMF. These findings warrant further investigation into how personality traits may help clinicians design targeted and effective interventions to reduce fatigability, and consequently lower the risk of adverse aging-related health outcomes.

